# Contemporary Theory and Research

**Published:** 1893-05-27

**Authors:** 


### CONTEMPORARY THEORY AND RESEARCH.

### VACCINAL IMMUNITY.

According to a report of M. Hervieux, before the Académie de Médecine, the bactericide state of the blood which constitutes vaccinal immunity is due to the passage into the blood of the products of secretion of the vaccinal bacteria. The bacteria themselves are destroyed by the leucocytes. Vaccinal bacteria have so great a power of proliferation that one alone suffices to give immunity. According to this theory the decrease of vaccinal immunity is explained by the renewal of the tissues and humours in the organism. According as this renewal is more or less complete the inoculations will remain either wholly or partially effective. The activity of the renewal of the humours in a child, and its slow action in an adult or aged person, explain why vaccinal immunity subsists for a less period in a child than in older persons. Practically, revaccination constitutes the only means of measuring the degree of vaccinal immunity which still subsists.

### EUCALYPTOL.

Bichlorate of eucalyptene appears under the form of a crystalline substance, insoluble in water and glycerine. According to experiments on animals and clinical observations made by M. Lafage and M. Sully at the Codim Hospital in Paris, bichlorhydrate of eucalyptene, while possessing scarcely any toxic properties, is an antiseptic modifier of expectoration, &c., and can be employed with advantage in chronic bronchitis, pneumonia, and other affections of the respiratory organs. It can be administered in cachets of 25 centigrammes, in the intervals between meals to the extent of a daily dose of 1 or 1½ grammes. Children take it readily under the form of saccharate in water or milk.

### THE CONTAGION OF INFLUENZA.

Writing in the *Medical Magazine*, Dr. Fitzgerald says, "All procurable evidence points to some air-borne contagium which naturally first impresses the parts immediately exposed to its influence, namely, the respiratory organs, the mucous membranes of the eyes and nose; and lastly, the intestinal tract. . . . If the contagium of influenza be aërial, it means an atmosphere laden with pathogenic spores in which we are immersed, and which must profoundly modify and exaggerate every co-existent malady. . . . It is this universal contamination of the surrounding atmosphere which renders compulsory notification and enforced isolation so hopeless a measure, although, as personal infection is not only possible but probable, partial or home isolation is not without its uses. It must have been some instinctive perception of this universal influence which led the Italians to name it 'Influenza'—the influence, for it certainly influences both the sick and the healthy; but instead of imputing it to spores or miasmata, they attributed it to the stars."

